# Effect of Mould Orientation on the Field-Dependent Properties of MR Elastomers under Shear Deformation

**DOI:** 10.3390/polym13193273

**Published:** 2021-09-25

**Authors:** Muntaz Hana Ahmad Khairi, Saiful Amri Mazlan, Ubaidillah, Nur Azmah Nordin, Siti Aishah Abdul Aziz, Nurhazimah Nazmi

**Affiliations:** 1 Engineering Materials and Structures (eMast) iKohza, Malaysian-Japan International Institute of Technology, Universiti Teknologi Malaysia, Jalan Sultan Yahya Petra, Kuala Lumpur 54100, Malaysia; hana5700@gmail.com (M.H.A.K.); nurazmah.nordin@utm.my (N.A.N.); sitiaishah.aa@utm.my (S.A.A.A.); nurhazimah@utm.my (N.N.); 2 Advanced Vehicle System (AVS) Research Group, Universiti Teknologi Malaysia, Jalan Sultan Yahya Petra, Kuala Lumpur 54100, Malaysia; 3 Mechanical Engineering Department, Faculty of Engineering, Universitas Sebelas Maret, Jl. Ir. Sutami 36A Kentingan Jebres, Surakarta 57126, Indonesia

**Keywords:** magnetorheological elastomer, orientation, alignment, anisotropic, isotropic, silicone oil, plasticizer, mold, stiffness, 45°, modulus, magneto-induced, MR effect

## Abstract

In this study, magnetorheological elastomer (MRE) was fabricated using an electromagnetic device with a new configuration mold at the orientation of 0°, 45° and 90°. This new curing concept enhanced the alignment of carbonyl iron particles (CIPs) within the silicone matrix in the presence of silicone oil (SO) during solidifying, by eliminating air gaps to prevent magnetic flux losses. Using a mold made of steel, which is a magnetic material, the mold functions as a guide for concentrated magnetic flux of 0.315 T to pass through the MRE sample. Scanning electron microscopy (SEM) was used to observe the surface morphology of the fabricated MRE samples particularly the alignment of the CIPs. The field-dependent dynamic properties of the MREs were measured using a rheometer. The analysis implied that the effectiveness of the MRE operating under shear deformation with this curing concept provided the highest magneto-induced modulus of 1.01 MPa when a 45° orientation mold is used, with relative magnetorheological (MR) effect value up to 918%, followed by 0° mold orientation with 0.79 MPa magneto-induced modulus and 646% relative MR effect. The high modulus properties offered by this MRE are believed to be potentially useful in industrial applications where a high range of stiffness is required particularly in the shear direction.

## 1. Introduction

Magnetorheological elastomer (MRE) has attracted much interest as the material can exhibit a desirable performance owing to the stability of magnetizable particles in a solid-based matrix [1,2,3,4]. MRE is a polymer composite, which mainly consists of a rubber matrix, magnetic particles as a reinforcement phase, and optional additives for acquiring specific properties such as increased stability and providing much more superior bonding between the matrix and the magnetic particles [5,6,7,8,9]. Depending on the curing process, it can be classified into two categories; isotropic MRE, which contains randomly distributed particles, and anisotropic or aligned MRE, which contained polarized particles following the direction of the magnetic field during the fabrication process [10,11]. Due to this fact, anisotropic MRE possesses closer gaps between the magnetic particles, as compared to that in isotropic MRE. This can lead to strong interactions between the particles, which make it much more sensitive towards the change in the intensity of the magnetic field causing a change in the stiffness of the material. The changes in the stiffness, normally in the range between 0.1 to 0.6 MPa, are called MR effect and it becomes a crucial parameter to evaluate the performance of MRE [12,13,14]. Correlation between particles distribution and MR effect have been supported by the numerical results of Xia et al. [6], who demonstrated that the particles’ distribution have significant effects on the MR effect. Moreover, according to Samal et al. [10], the sample dimension can also affect the distribution of CIPs in the MRE thus consequently affecting the MR effect.

Recently, MRE was developed to acquire a high MR effect with the addition of plasticizer [7,15,16,17] and by orienting the magnetic particles particularly carbonyl iron particles (CIPs) [18] or a combination of both approaches [19]. A plasticizer, typically a solvent, is added to a synthetic resin to produce or promote plasticity and flexibility of polymer materials. With this method, the MRE becomes softer and exhibits a higher relative MR effect, as a result of lower zero-field modulus or initial storage modulus [20,21]. Therefore, the interaction between magnetic particles and matrix phase during the fabrication process has been improved and at the same time, has modified the stiffness of MRE. Moreover, the plasticizer acts as a lubricant and allows molecular chains of rubber to glide easily during the curing process, thus decreasing the adhesiveness of a rubber matrix [22,23]. In other words, this advantage of plasticizer helps in aligning the process of the CIPs in an anisotropic MRE. As a result, the CIPs are aligned utterly following the direction of the magnetic field in the MRE.

The use of plasticizer was introduced by Lokander et al. [16], namely di-2-ethylhexylphthalate into isotropic nitrile rubber-based MRE. This study obtained a slight improvement in the relative MR effect of MRE; however, no quantitative data were shown by them. Furthermore, Chen et al. [17] studied the influence of mineral oil as a plasticizer on the shear storage modulus of anisotropic natural-based MRE. In their study, the plasticizer used was between 10 to 20 wt.% and the result revealed that the addition of 10 wt.% mineral oil provided an initial storage modulus of 1.4 MPa, and magneto-induced modulus of MRE was 0.23 MPa. Meanwhile, when the amount of mineral oil increased to 20 wt.%, the initial shear storage modulus and magneto-induced modulus of MRE became 0.9 and 0.7 MPa, respectively. In brief, the relative MR effects of MRE were at 21% and 78% when 10 and 20 wt.% mineral oil were added, respectively. For the case of silicone rubber-based MRE, Gong et al. [15] studied the effect of silicone oil (SO) in an anisotropic MRE. The addition of 15 wt.% SO exhibited a relative MR effect up to 400% with the range of storage modulus was between 0.25 to 1.25 MPa, at 1 T. The anisotropic MRE contained 70 wt.% CIPs and was cured under the magnetic field of 1.5 T. Therefore, the addition of plasticizer has proven to enhance the MR effect of the MRE.

Other than the use of plasticizer, the MR effect can be enhanced by orienting the CIPs in the MRE during the curing process; though the study is rather limited. Previously, the conventional anisotropic MRE had an orientation of 0° of CIPs to the *y*-axis [15,24]. However, the study was evolved by focusing on the various orientations of CIPs in anisotropic MRE. A study on CIPs orientation other than 0° anisotropic MRE was conducted by Boczkowska et al. [18]. Polyurethane-based MRE was developed with the same weight fraction of 60 wt.% magnetic particles with different particle orientations including 0°, 45°, and 90°. The experiment revealed that the largest MR effect was obtained at 45° to the direction of the magnetic field. The order of the values of the MR effect versus the orientation was 90° < 0° < 45°. However, the study did not show how they obtained CIPs orientation of 0°, 45°, and 90° during the fabrication process; moreover, no microstructure images of the MRE with 0°, 45°, and 90° were disclosed.

Rather than individually focusing on the use of plasticizer and CIPs orientation, MR effect can also be improved by combining both approaches. Tian et al. [25] introduced SO plasticizer to the fabrication of anisotropic silicone rubber-based MRE with 45° of CIPs orientation by rotating the non-magnetic mold to 45° relative to the applied magnetic field that is in the vertical direction. The team found that the MR effect in one shear direction was at 580% compared to that in the opposite direction, which was at 480%. In that study, 15 wt.% SO was used as a plasticizer with 70 wt.% of CIPs as magnetic particles in the fabricated MRE. In another study, Yao et al. [19] developed a new model to describe the shear behavior of MRE with particle chain’s orientation including 0°, 45°, and 90°. The model portrayed micro-mechanical interaction between particles in the MRE, based on the theory of magnetic dipoles and magnetic field coupling between particles that aligned in the chain. The results of the model revealed that the MR effect of MRE has a strong dependence on the orientation of the particle’s chains and 45° of CIPs orientation resulted in the highest MR effect [19]. However, in experimental verification, the selection of a low percentage of CIPs and SO, particularly 33 wt.% and 0.4 wt.%, respectively, resulted in a low relative MR effect of MRE, which was 115%. The low MR effect of MRE showed that the composition of CIPs and SO as plasticizer was crucial and must be vividly considered to fabricate MRE with the expectation of a higher MR effect. This is despite the fact that anisotropic MRE was developed to acquire a high MR effect with the addition of plasticizer [15] and in various CIPs orientations [18] or a combination of both approaches [19,25]. Current developed anisotropic MREs have MR effect below 600%, whereas if the MR effect can be achieved at more than 600%, it can be beneficial to applications that require a high MR effect, especially in the shear direction. The obtained MR effect which is below 600% of MRE was probably due to the limitation of the curing device, which led to the flux losses during the curing process, hence affecting the CIP’s alignment.

Generally, the concept of a curing device that other researchers used in the past for anisotropic curing mostly was made from a non-magnetic materials slab’s mold that was divided into two parts, top and bottom [2,19,25,26]. The sample was placed in the middle, sandwiched between the upper and bottom mold. In this design, the non-magnetic mold has not properly guided the magnetic flux to flow into the MRE. Moreover, this design might create high magnetic flux losses due to the air gap between the mold and the coil, resulting in insufficient magnetic flux that penetrated the MRE sample during the curing process, consequently, affecting the alignment process of CIPs. As stated by Tian et al. [25], the measurements of flux density were taken outside of the non-magnetic mold and it was found that the magnetic flux density was more than 0.3 T to align the particles particularly at 45° within the matrix phase. However, it is unsure that the same 0.3 T was flowing inside the mold to align the magnetic particles in the MRE, due to their curing device design; the magnetic flux might be lost to the air. Hence, the alignment process of CIPs in an MRE at a specific degree might have deteriorated that subsequently affecting the corresponding MR effect as well.

On the other hand, another anisotropic curing setup was presented by Samal et al. [27], where the MRE sample was designed cylindrically located on the top of the curing device. The simulation of magnetic distribution was reported to be different at the top, middle and bottom of the MRE sample, and the average value of magnetic induction concluded to be around 0.3 T after considering the ranges from the top level to the bottom of the MRE sample. The mentioned studies used plasticizer and orientation of CIPs in an MRE could result in improvement of the MR effect. However, until now the maximum modulus increase for the MRE has been reported to be up to 0.6 MPa with magnetic particles at about 70 wt.% [27] probably due to the poorly aligned CIPs inside the MRE caused by the insufficient magnetic flux penetrating the MRE during the curing process. Thus, it is an opportunity to provide a uniform magnetic flux to ensure the particles are aligned (assemble into chain-like microstructures) before being locked up in the rubber-based matrix as a cured MRE in a solid-state [1].

The main technical contribution of this work is to obtain MRE (employed with SO) with a high MR effect of more than 0.60 MPa magneto-induced modulus for widely industrial application. To achieve that, the magnetic response between the CIPs must be improved by enhancing the alignment of CIPs. This can be done by modifying the existing anisotropic curing device by using a “new configuration magnetic flux mold” that consists of a cylindrical mold which is inserted into the magnetic coil without an air gap to prevent magnetic flux loss, and the molds that are made from a magnetic material, which functions as guidance for the concentrated magnetic flux flow in the MRE mold. MRE sample (1 mm thickness) was designed to be sandwiched between the top and bottom of mold for enhanced alignment and uniform magnetic distribution when the concentrated magnetic flux was passing through the MRE during the curing process. The molds were employed with 0°, 45°, and 90° orientations (to the *y*-axis) to investigate the effect of different orientations towards the MR effect. After fabricating the proposed MRE samples, observations via scanning electron microscopy (SEM) are made to observe the particles’ alignments in the samples. Subsequently, the field-dependent dynamic viscoelastic properties of the MREs were measured using a rheometer. The measured results were compared, and the effectiveness of the proposed MRE was validated to determine the enhancement of the magneto-induced and relative MR effects. It should be noted here that to the best of the authors’ knowledge, there is no MRE using this magnetic flux mold’s concept for orientation specifically at 0°, 45°, and 90° so far.

## 2. Materials, Fabrications, and Methods

### 2.1. Materials

The elastomer used as a matrix material for the manufacture of MREs is silicon rubber base, type RTV-two NS625 supplied by Nippon Steel. It is physically a white flowable liquid that would have a hardness of 22–50 Å after the completed curing process. The magnetic particles, CIPs are from BASF, Germany with an average size of 6 µm. The anisotropic curing condition using the new concept of mold where the particles are configured at 0°, 45°, and 90° orientations as well as isotropically. Fabricated MRE samples were using 70 wt.% of CIPs with 15 wt.% of SO, followed by 15 wt.% of silicone rubber. SO plays an important role to soften the matrix which can reduce the zero-field storage modulus of MRE and enhanced the maximum stiffness change of MRE. Moreover, SO should improve the adhesive properties of the particles on the matrix surface [27]. It should be noted that one sample without SO was prepared for comparison, and viscosity test for uncured sample will be carried out to determine the MRE viscosity before the solidification process.

The required amount of silicone rubber, SO, and CIPs were weighed accordingly. The materials were mixed thoroughly and uniformly using a mechanical stirrer for 20 min. Then, the curing agent was added and the mixture was stirred again for 2 min. This is to allow the curing process to occur and transform the paste-like sample to become solid. Next, the final mixture was poured into a customized steel mold (0°, 45°, and 90° molds) with the size of 1 mm in thickness and 70 mm diameter and left for solidification with the absence of magnetic field, particularly for the isotropic condition. For the 0°, 45°, and 90° orientations mold, the mixture was cured with the presence of a magnetic field in terms of current applied from electromagnetic circuit. For the isotropic condition, the mold of 0° was used but without a magnetic field applied during the respective curing process so that the magnetic particles were homogeneously distributed in the MRE. All MRE samples are labelled as MRE 0°, MRE 45°, MRE 90° and isotropic MRE.

### 2.2. Curing Device and Simulation of Magnetic Flux

**Figure 1:**
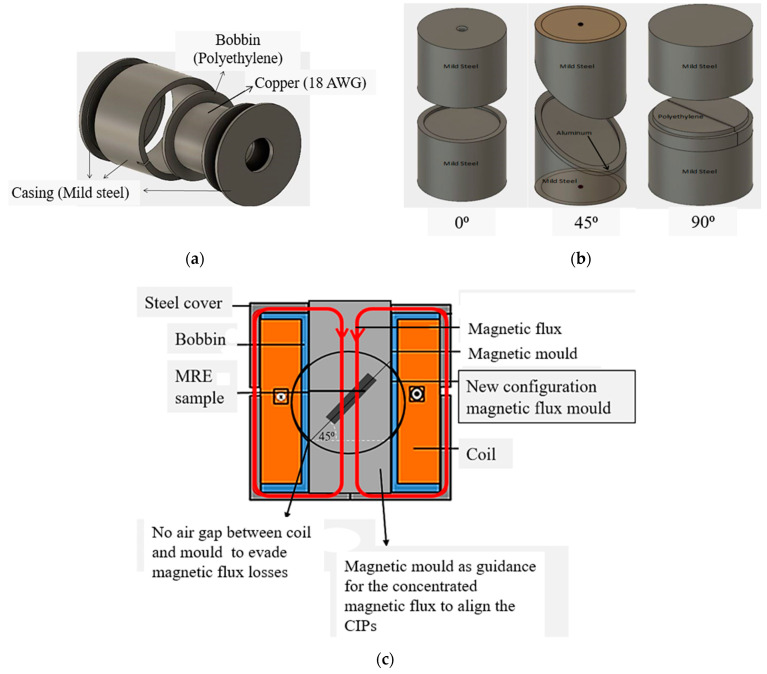
The anisotropic curing device (**a**) design of casing (**b**) design of mold (**c**) schematic diagram.

The curing device was used with a new concept of mold configuration to generate a focused-guided magnetic field with the electromagnetic circuit in the sample during the curing process of the anisotropic MRE with different mold orientations. In general, the highest magnetic field strength with a certain alignment of magnetic fluxes that passed through the sample is the foremost requirement to form anisotropic MRE. In other words, the new configuration of electromagnetic circuit and mold can generate the maximum magnetic field strength into the sample and at the same, the magnetic field can be controlled via the electromagnetic circuit. This new curing device was comprised of coil, casing, separator and mold. The coil, 18 AWG with a diameter of 1.11 mm, was made of copper and was rolled up to the non-magnetic bobbin (polyethylene) with 2270 turns. The casing and the mold are made of mild steel, while the separators, which are used for directing the magnetic field into the mold, are made of polyethylene for mold 90° and aluminum for mold 45°. The schematic diagram of the modified curing device is illustrated in Figure 1 and details of the input parameter of the components are listed in Table 1.

**Figure 2:**
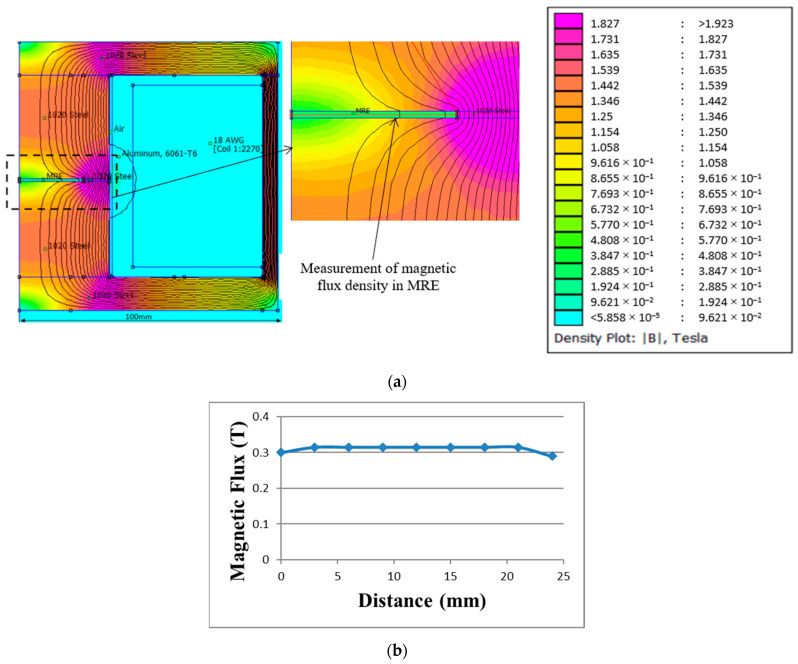
Simulation of magnetic flux density using FEMM: (**a**) distribution and (**b**) the value of magnetic flux across the MRE sample.

Finite element method magnetics (FEMM) was performed during designing the mold concept to measure the magnetic flux density that passes through the sample as illustrated in Figure 2 based on input parameters in Table 1. It can be observed that the distribution of magnetic flux density, shown in red (darkest shade), signifies the presence of maximum magnetic flux in that region. The green-yellow (lighter shade) color signifies the presence of moderate magnetic flux. The minimum magnetic flux region is denoted by the blue color (white or no shade) especially at the casing and surrounding region. The simulation was performed to obtain at least 0.3 T of magnetic flux density to align the particles within the matrix phase as stipulated by Tian et al. [25] and Samal et al. [27]. As a result of the simulation, 0.315 T of magnetic flux density, as shown in Figure 2b, was achieved at applied current of 0.2 A. The current was adjusted accordingly to maintain the same value of magnetic flux density for 45° and 90° molds. The actual measurement of the magnetic flux density at the fabricated molds was carried out via a specific hole on the casing wall, using a tesla meter.

**Figure 3:**
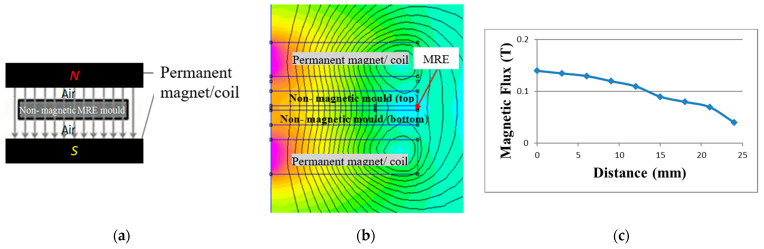
Simulation of magnetic flux density using FEMM of commonly used in previous studies: (**a**) Schematic drawing of the curing setup (**b**) distribution (**c**) value of magnetic flux across the MRE sample.

As for the comparison, the concept of mold that other researchers used in the past for anisotropic curing was reproduced through simulation as shown in Figure 3 [2,19,25,26]. As observed through FEMM, the mold yielded magnetic flux density of 0.1 T as opposed to the new configuration, where it achieved 0.315 T when same setup (design, number of coil turns, and current input) was applied. Moreover, the values of magnetic flux density were uneven across the MRE that deteriorated alignment of CIPs inside the MRE.

**Figure 4:**
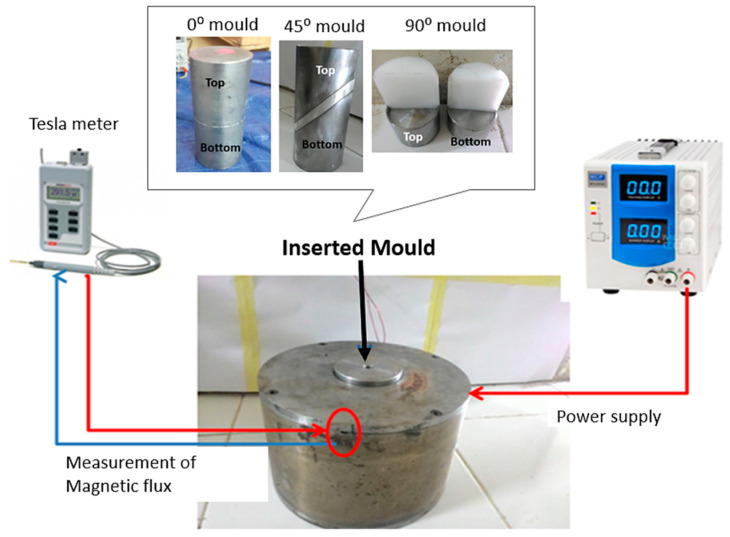
The setup of a curing device for anisotropic MRE with various mold orientations was inserted.

Figure 4 shows the experimental set-up used for curing process of the anisotropic MRE with 45° mold that cured under the presence of magnetic flux density (0.315 T). The same coil was used to fabricate MRE 0°, 45°, and 90° molds by placing them individually at the center of the electromagnetic coil. The location of the flux density measurement at a specific space inside the chamber was also disclosed.

**Figure 5:**
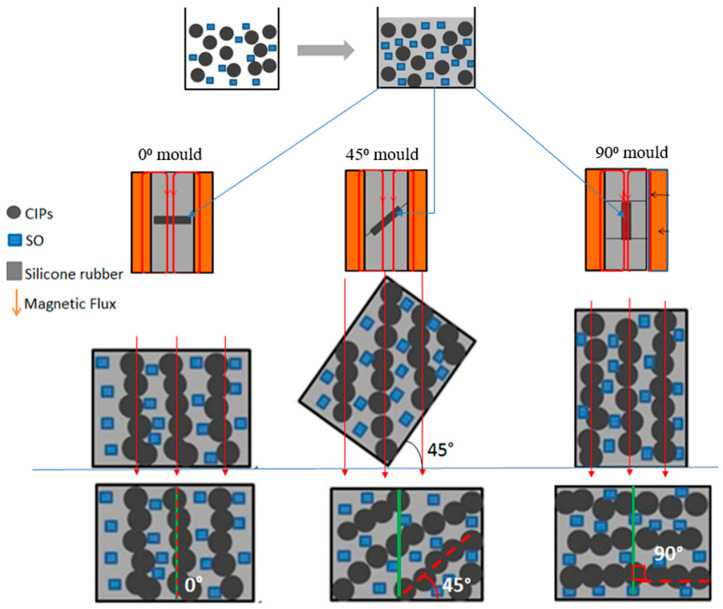
Illustration of CIPs orientation with 0°, 45° and 90° to the *y*-axis during curing process.

Meanwhile, Figure 5 shows an illustration of the alignment of CIPs in the matrix phase during the solidification process in the molds of 0°, 45° and 90° to the *y*-axis. The mold was used as guidance for the concentrated magnetic flux to flow parallelly from top to bottom parts of the mold. The high magnetic flux that passed through the MRE sample, and sandwiched between the mold’s parts, resulted in the enhancement of the alignment of CIPs within the silicone matrix phase. On the other hand, another anisotropic curing setup was introduced by Samal et al. [27], where the MRE samples were designed cylindrically located on the top of the curing device with length of 20 mm and a radius of 5 mm in contact, by its lower and upper surface, with copper slabs in the circuit of the magnetic field. The magnetic distribution was reported to be different at the top, middle and bottom of the MRE sample. In this work, as mentioned in the previous text, the MRE sample (1 mm thickness) to be sandwiched between the cylindrical magnetic mold that is inserted into the electromagnetic coil for better alignment and uniform magnetic distribution throughout the MRE sample. The thickness of the sample was designed to be 1 mm, suitable for rheology test using a rheometer.

### 2.3. Magnetic Properties Measurement and Morphological Observation

The magnetization curves were measured at room temperature using Vibrating Sample Magnetometer (Micro Sense, EZ series). The working principle of using VSM was started by placing the MRE sample at a constant magnetic field. The MRE sample was protected by wrapping it with a polytetrafluoroethylene (PTFE). A constant magnetic field would magnetize the sample, and the magnetic domains of the particles vibrated and tended to align, along the direction of the magnetic field. The magnetic dipole moments of the sample created a magnetic field around the sample, and the changes of the magnetic fields were sensed by a coil. The magnetization of the sample depended on the strength of the constant magnetic field. The magnetic field was ranging from −1250 to 1250 kA/m and the graph of magnetization (M) versus magnetic field strength (H) was then generated. The important outputs generated from the tests including magnetic saturation (Ms), coercivity (Mc) and retentivity (Mr), which determined the magnetic behavior of the MRE materials.

The morphological characterization of the MRE samples was observed under Low Vacuum Scanning Electron Microscopy (LVSEM, JEOL JSM-IT300, Tokyo, Japan) at an acceleration voltage of 15 kV up to 1000 times magnifications. The LVSEM is used to scan the cross-section surface of samples to create an image using focused beam electrons. The MRE samples were coated with a gold layer on the surface to avoid any charging during observation. Then, a sample was mounted rigidly to a specimen holder using a conductive adhesive and it was placed in the sample chamber. The emitted electron beam scanned the cross-section of the samples, where the electrons in the beam interact with the sample, producing various signals that can be used to obtain information about the surface topography and composition. The determination of the elements in MRE were confirmed through energy-dispersive X-ray fluorescence (EDX, JEOL, JED-2300, Tokyo, Japan).

### 2.4. Rheological Properties Test

In the rheology test, all samples were examined in uncured and cured conditions which were related to viscosity and viscoelastic investigation, respectively. The viscosities of the uncured MREs with and without SO were measured using a rotational rheometer (Physica MCR 302 from Anton Paar, Graz, Austria). The samples were tested at 25 °C controlled by the Viscotherm VT2 (Anton Paar, Graz, Austria) at a shear rate of 0.001–10%, and the gap between the oscillatory disk was fixed at 1 mm. The dynamic viscoelastic properties of the cured MRE samples were evaluated using the oscillatory rheometer MCR 302. The rheometer MCR302 was equipped with the current controller MRD70 to generate the required magnetic field through the system. The measurements were performed by sandwiching the MRE samples between a rotary disk and a parallel base plate, PP20 (Anton Paar, Graz, Austria). The dimensions of the samples were 20 mm in diameter and 1 mm thick. The samples were tested under different flux densities from 0 to 800 mT with different frequencies at 1, 5 and 10 Hz. For the effect of strain amplitude sweep, the strains were fixed at 0.01, 0.1, and 1%.

## 3. Results and Discussions

### 3.1. Magnetic Properties

**Figure 6:**
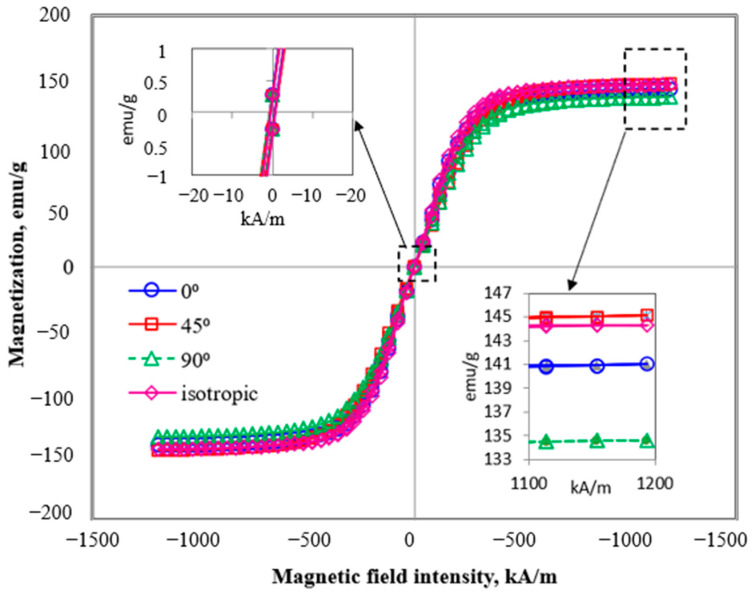
Hysteresis loops of MRE samples at 0°, 45°, and 90° orientations of mold, and isotropic MRE.

The magnetization versus magnetic field (known as the hysteresis loop) was carried out to investigate the effect of magnetic characteristics of MRE with various CIPs orientations. Figure 6 shows the magnetic field strength (kA/m) versus magnetization of MRE samples with various SO concentrations in the form of hysteresis loops. In general, a magnetic moment (or magnetic dipole moment) is a measurement of a specific object to interact with the applied magnetic field. The object will tend to be arranged so that its magnetic moment vector aligns with the lines of the magnetic field. The hysteresis curve is obtained by increasing the magnetic field in both negative and positive directions. The magnetization will reach its saturation, known as magnetic saturation (Ms) with a further increase in the applied magnetic field, H.

Figure 6 illustrates the magnetization curves of MRE samples with various orientations. It is noted that those MRE samples have contained 15 wt.% SO. Generally, the MRE samples with various orientations of CIPs exhibited narrow hysteresis loops indicating the soft magnetic characteristics of MRE since CIPs were used as reinforced magnetic particles in the rubber matrix. As observed in Figure 4, the magnetic hysteresis loop of MRE samples possesses a similar trend either at 0°, 45°, or 90° mold orientations and the *M_s_* values were noted to be in the range of 135–145 emu/g. A small variation in *M_s_* values (±10 emu/g) might be due to the selected area of the sample for the magnetic properties test that consisted of various sizes of CIPs in the range of 1–10 μm. Thus, it is uncontrollable to acquire testing samples with the same distribution of CIPs. In addition, the retentivity, *M_r_* and coercivity, *H_c_* values of the MRE samples were not influenced by the variations of CIPs oriented as shown in Table 2. The *H_c_* and *M_r_* values were in the range of 8–8.3 and 0.27–0.29, with the deviation values were just around Δ0.3 and Δ0.02, respectively. *M_r_* stands for magnetic remanence of the magnetic material, which refers to the amount of magnetization that remained in the MRE after the applied magnetic field was removed. In the meantime, the *H_c_* is a measurement of the magnetizing force required to drive reverse magnetization once it has been saturated. These data show that the MRE with mold orientations at 0°, 45° and 90° to the *y*-axis and isotropic did not affect the magnetization behavior of the MRE. On the other hand, the value of *M_s_* was dominantly dependent on the content of the CIPs within the MRE sample [28].

### 3.2. Morphology of the MRE

**Figure 7:**
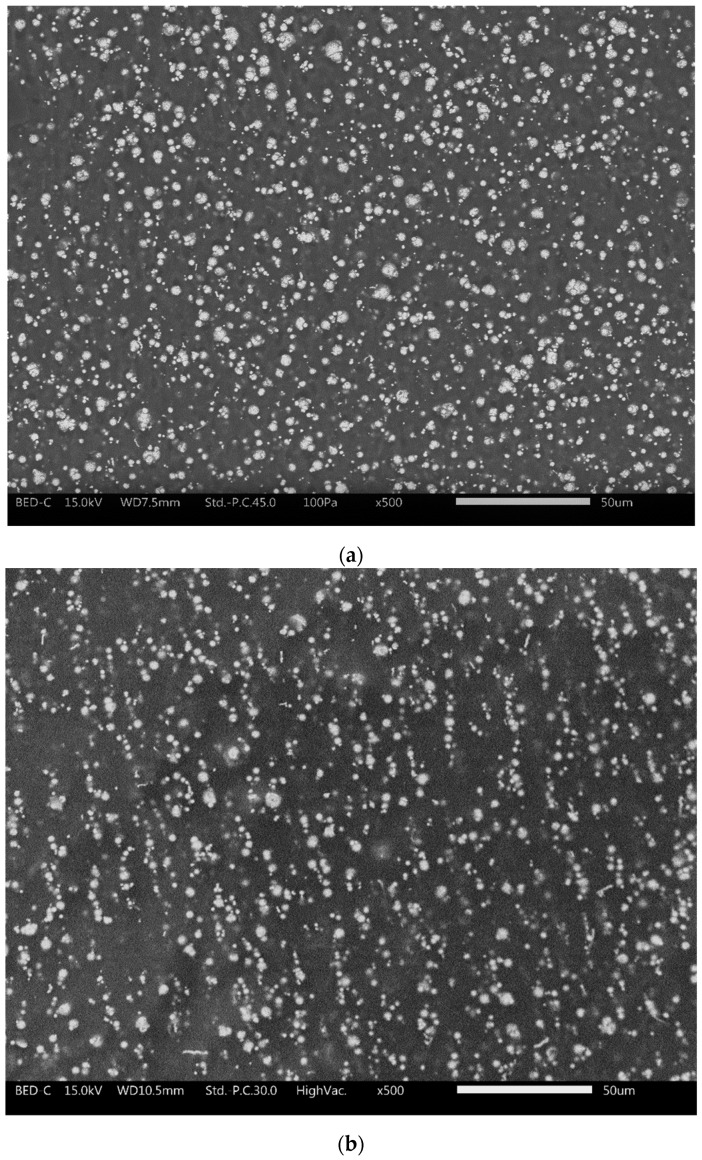

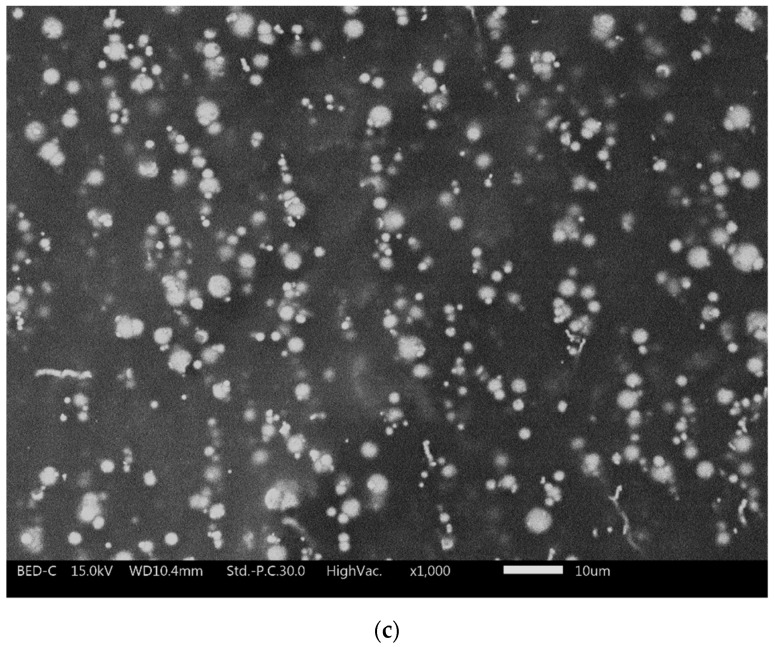
SEM images of MRE (**a**) isotropic at 500× magnifications (**b**) anisotropic at 500× magnifications (**c**) anisotropic at 1000× magnifications.

The images of the cross-sectioned morphology of MRE samples are presented in Figure 7. It can be observed that for the isotropic MRE, the CIPs are dispersed randomly in the matrix phase, as shown in Figure 7a. Meanwhile, Figure 7b,c shows the alignments or chain-like structure of the anisotropic MRE with magnification of 500 and 1000 times, respectively. The CIPs are observed to be aligned forming the chain-like structure of particles, as a result of following the direction of magnetic field during curing process. According to Samal et al. [27], particles become magnetized and assembled into chain-like structures in a uniform magnetic field, due to magnetic dipole-dipole interactions, thus tend to align with the direction of external field. In this work, the new configuration magnetic flux mold concept generated a focused magnetic field into the mold and towards the pre-polymer MRE during the curing process. The interaction and attraction between the CIPs led them to align with the flow of magnetic field. As the curing reaction took place, the CIPs’ chain-like structure became fixed and locked in the aligned manner in the matrix. As mentioned in the previous sub-section, the FEMM simulation shows that the concentrated and uniform magnetic flux of 0.315 T passed throughout the MRE sample, thus enhancing the alignment of the CIPs, consequently enhancing the MR effect that will be discussed in the rheology part in the next sub-section.

On the other hand, the distribution of the distance between adjacent particles to the resultant rheological properties of MRE was proposed by Suo et al. [29] using a magnetic dipole model based on chi-squared distribution. The mathematical models have considered the distance between magnetic particles and their magnetic dipole have affecting the rheological behaviour of the MRE. In another study, the correlation between the orientation of particles to the rheological properties of MRE was reported by Davis [30], whose study employed the magnetic dipole theory to compare the rheological behaviour of MRE before and after the dispersion of the magnetic particles. The study discovered that the rheological properties of the material were enhanced after the homogeneous dispersion of the magnetic particles was achieved. Additionally, the 3D images internal structure of the MRE were also investigated, and the simulation result revealed that the distribution of filler particles within the volume, in both isotropic and anisotropic distribution have a significant effect on the properties of MRE [31].

**Figure 8:**
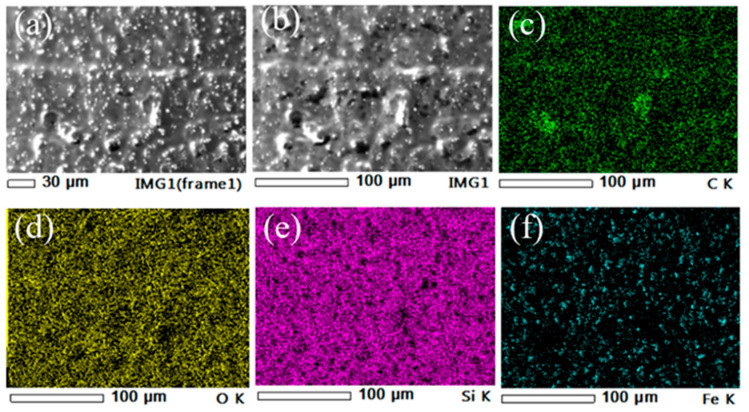
EDX mapping analysis of MRE sample consisting of (**a**,**b**) EDX region, (**c**) Carbon, green (C), (**d**) Oxygen, yellow (O), (**e**) Silicone, purple (Si) and (**f**) Iron, blue (Fe) elements.

On the other hand, the elemental composition of MRE was identified through Energy Dispersive X-ray Analysis (EDX) mapping analysis as shown in Figure 8. The EDX mapping images traced the elements of Si, O, and C in both silicone rubber and SO. Meanwhile, the element Fe represented the CIPs in the MRE. Therefore, the mapping results were confirmed with the LV-SEM microstructure observations and MRE formulation.

### 3.3. Rheological Properties: Effect of Mold Orientation

**Figure 9:**
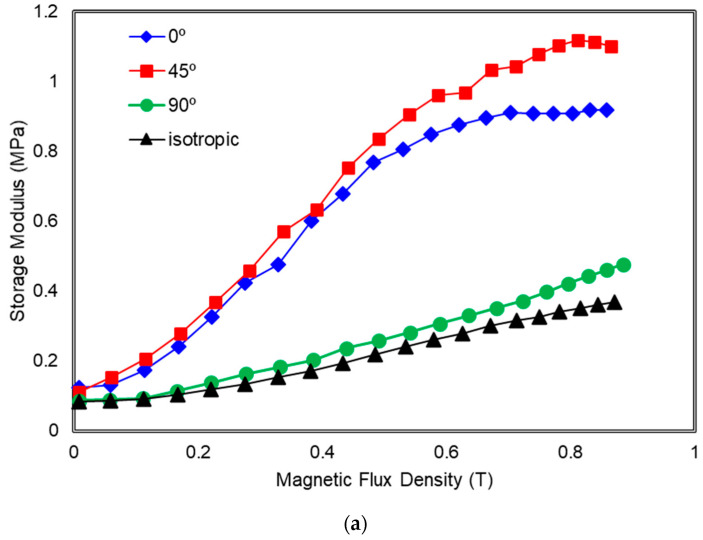

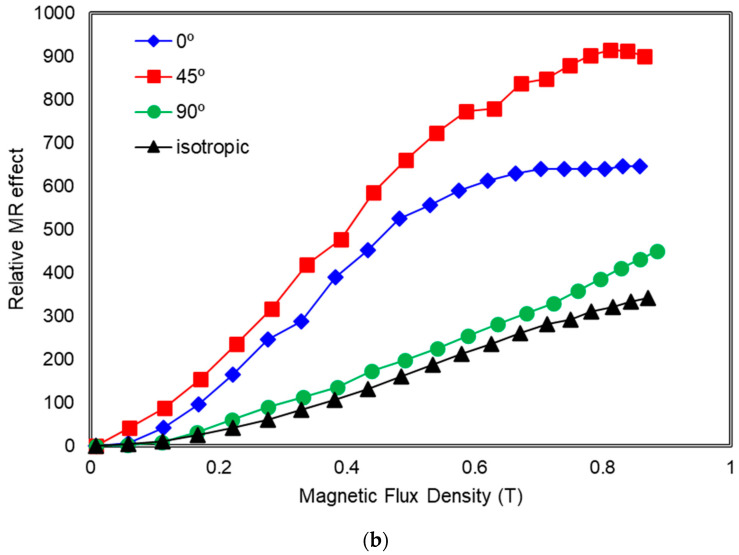
MRE with the various orientation mold of 0°, 45°, 90° and isotropic (**a**) Storage modulus (**b**) Relative MR Effect as a function of magnetic swept.

The effects of mold orientations of 0°, 45°, and 90° on the storage modulus and relative MR effect of MRE under different magnetic fields are shown in Figure 9. The initial storage modulus, G_0_ and MR effect of MRE samples including isotropic and all orientations are summarized in Table 3. From the table, it is observed that the G_0_ of the MRE samples is not much influenced by the orientation of mold whether those are in 0°, 45° or 90° to the *y*-axis, and also in isotropic conditions. Indeed, the fabrication of MRE samples with 0°, 45°, and 90° mold orientation using the new configuration of magnetic flux mold enhanced the alignment of CIPs by providing more magnetic flux flows into the samples during the curing process. This consequently increased the MRE’s maximum value of stiffness due to particles’ strong interaction and providing a higher range modulus of MRE. In addition, the orientation of CIPs and their movement in MRE were much easier with the help of 15 wt.% SO as a plasticizer and the CIPs were denoted closer to each other during the curing process, for all orientations. However, it can be seen from the experimental results that the MR effect of MRE maximizes its value for the orientation of using 45° mold. The order of maximum modulus (G_max_) and MR effect of MRE versus orientations of CIPs are 45° > 0° > 90°, which is consistent with the findings of Boczkowa et al. [18] and Yao et al. [19]. Furthermore, the resultant MR effect of the MRE with 45° CIPs oriented is much larger due to the enhancement of the orientation of CIPs via new flux configuration mold during the pre-structure process of the material. The magneto-induced modulus and relative MR effects have reached 1.01 MPa and 918%, respectively. Both have exceeded the results from Tian et al. [26] and Yao et al. [19] who depicted a value of 0.3 MPa and 0.59 MPa for the magneto-induced MR effect of MRE, respectively. Meanwhile, MRE with mold 0° and 90° to the *y*-axis and with isotropic distribution exhibit a lower value of relative MR effect which are 646%, 433%, and 343%, respectively.

Under the influence of the magnetic field, the orientation or chains of CIPs tend to be set according to the direction of the magnetic field to minimize the Zeeman energy, which involves the displacement of the particles. Because the CIPs are restrained by interactions at the interface between the particle-polymer phase and embedded in the elastomeric matrix, their displacements were introduced by additional shear stress into the matrix, especially at the orientation of 45°, causing an increased in stress concentration of the matrix and between the CIPs, consequently increased the stiffness of the whole MRE [32]. This can be attributed to the fact that the resultant MR effect of MRE is much higher for the orientation of 45°, followed by 0° and 90° as shown in Figure 9b. It also is noted that the lowest magneto induced, ΔG and relative MR effect have been obtained for MRE that has scattered CIPs (isotropic MRE).

**Figure 10:**
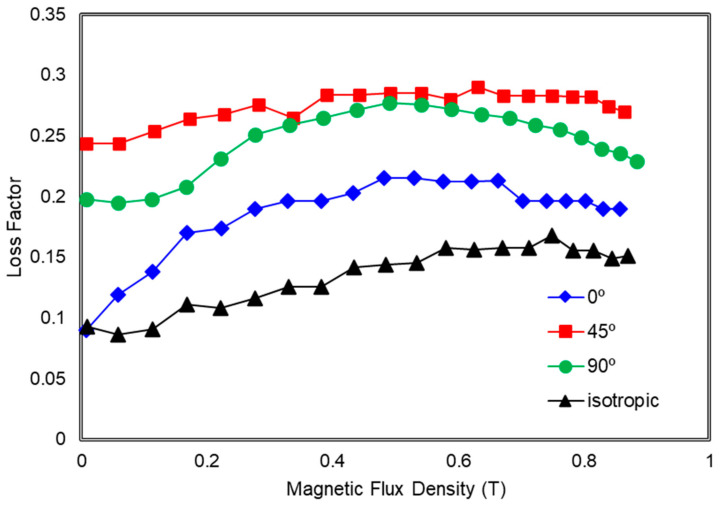
Loss Factor of MRE with the various orientation mold of 0°, 45°, 90° and isotropic as a function of magnetic swept.

Figure 10 shows the loss factor of MRE samples with various orientations of CIPs under different magnetic fields. As can be seen from the figure, particularly below 0.2 T, all MRE samples with 0°, 45°, and 90° of mold orientations show a low value of loss factor with the isotropic MRE exhibited the lowest value, around 0.1 MPa. However, as the magnetic flux density increased up to 0.6 T, MRE with 45° orientations of mold has the highest loss factor which is more than 0.25 MPa due to it giving the highest loss modulus compared to other orientations. Wang et al. [33] revealed that the loss factor or damping of the MRE is mostly caused by the interfacial friction between the magnetic particles and the rubber matrix.

It is noted that the material that exhibits higher storage modulus would also possess higher loss modulus and higher loss factor. Therefore, rubber-based material such as MRE is normally used for damping material. For the current research, it can be observed that the interaction of matrix and particles is also relatively weak at 45° orientation of mold thus, the sliding energy dissipation is produced easily under the applied shear stress. This phenomenon occurred when the magnetic particles are arranged at 45° in the MRE and the shear force is in a horizontal direction; the force generated between the particles would increase the energy dissipation, which is caused by the interfacial sliding hence increasing the energy loss [33]. However, when the higher magnetic field is applied to the MRE sample regardless of the mold orientation, because of the strong interaction force between the particles and the rubber matrix, the sliding displacement can be decreased. As a result, energy dissipation and the loss factor are lowered. Furthermore, due to the interaction between the magnetic particles, the rubber matrix is more restricted at higher applied magnetic fields. As a result, the molecular rubber’s energy dissipation is decreased.

### 3.4. Rheological Properties: Effect of SO

**Figure 11:**
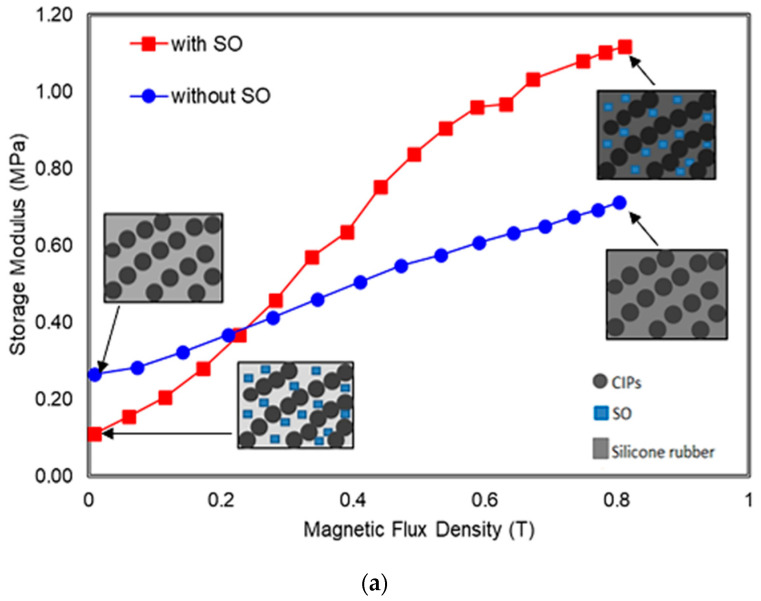

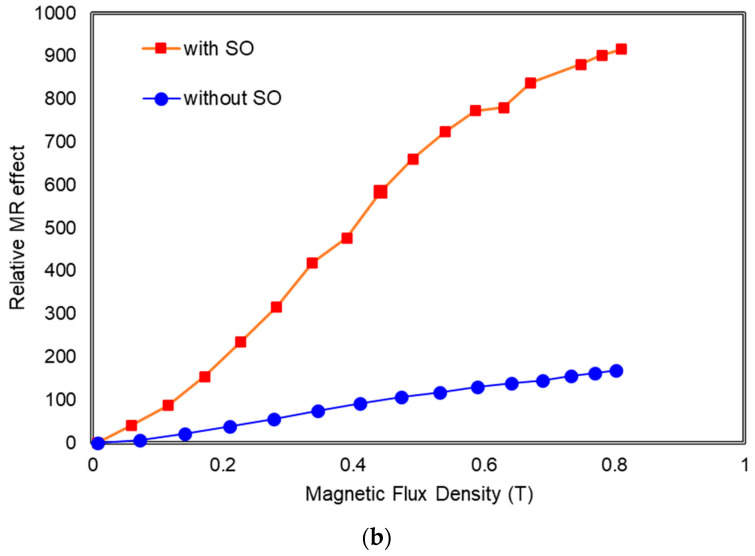
MRE with orientation mold of 45° with and without SO (**a**) Storage modulus and (**b**) Relative MR effect as a function of magnetic swept.

The effect of SO content on the storage and relative MR effect under different magnetic fields of MRE 45° samples are shown in Figure 11. Meanwhile, the related initial storage modulus and MR effect data for each sample are summarized in Table 4.

**Figure 12:**
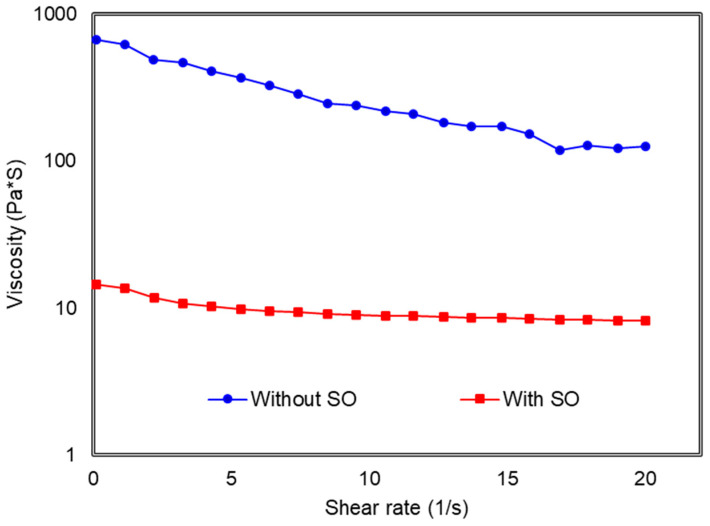
The viscosity of the uncured MRE samples with and without SO under the different shear rates.

It can be observed that both magneto-induced (ΔG) and relative MR effect of the MRE 45° sample increased with the magnetic field; however, the MRE effect increased greatly with the sample with SO. The maximum storage modulus (G_max_) for the MRE with SO reaches the maximum state at 0.8 T as shown in Figure 11a that was presented with the schematic micrograph. This mechanism has demonstrated how the increased magnetic field has stiffened the MRE sample that originally had different initial storage modulus, G_0_ which depending whether it was with or without SO. The grey intensity of the illustrated micrograph MRE becomes darker shade, showing the stiffer MRE as correspond to higher applied magnetic fields. In the presence of magnetic field, the addition of SO in the MRE 45° would make it stiffer as compared to MRE 45° without SO. Therefore, it can be briefly concluded that the magneto-induce storage modulus of the MRE 45° with SO particularly 15 wt.% SO exhibits 1.01 MPa with MR effect of 918%, which is 5 times bigger than that of MRE 45° without SO as in Figure 11b. This result exceeds the results from the literature with a depicted value of 0.6 MPa for the magneto-induced MR effect of MRE [19,25]. The associated improvement on the relative and magneto-induced MR effect of the MRE 45° by adding SO was achieved using the new configuration of magnetic flux concept, which is believed enhancing the alignment of CIPs by providing more magnetic flux flows into the MRE 45° which using the different approach as compared to Tian et al. [25] and Yao et al. [19]. Furthermore, the mobility and alignment of CIP’s were much easier during the curing process, especially when the content of SO used was 15 wt.%. This can be proven by determining the viscosity of the uncured MRE with and without SO as shown in Figure 12 under the different shear rates.

The viscosity of the matrix phase during the preparation of the MRE melt would determine the elasticity of the cured MRE. The most important processes in the fabrication of the MRE are the dispersion and homogeneous distribution of the CIPs in the rubber matrix. It is also well known that the matrix’s viscosity has a significant impact on the magnetic particles’ dispersion [34]. For the MRE without SO, the CIPs are difficult to disperse, and consequently mobility of the CIPs is restricted during the curing process. Meanwhile, the viscosity of the melt MRE decreased with the addition of 15 wt.% of SO then helps CIPs to align easily following the applied magnetic flux during the curing process, which resulted in the enhancement of the rheological properties of the MRE 45°. Figure 12 also depicted the increment of shear rate has reduced the viscosity of both MRE 45° samples. The molecular chain structures in the rubber matrix have experienced breakdowns upon applied shear stress and need time to recover hence reducing the crosslink density of the rubber that lowering the viscosity of the uncured MRE [35]. Associated with lower initial storage modulus and improved interaction between aligned CIPs with the addition of SO, the MR effect of MRE at 45° orientation mold has been improved [19] upon using a new configuration magnetic flux mold.

### 3.5. Rheological Properties: Effect of Strain Amplitude and Frequency

**Figure 13:**
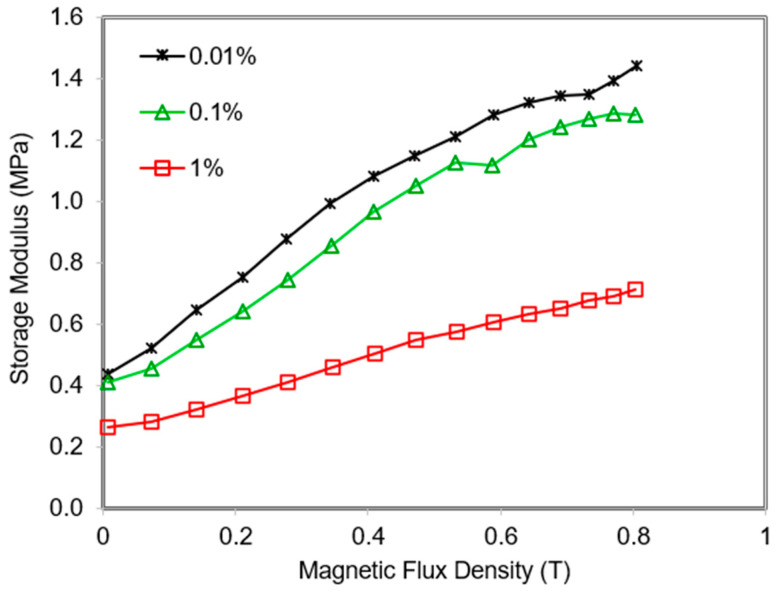
MRE 45° at various amplitude strains as a function of magnetic swept.

The magnetic field-induced modulus changes of the MRE 45° at three different strain amplitudes are shown in Figure 13. When the shear strain amplitude is 0.01% and 0.1%, the initial field modulus of the sample is almost the same. However, as the strain amplitude increases to 1%, the initial field modulus decreases from 0.38 MPa to 0.34 MPa. This is due to the sliding effect between particles and matrix increased during the motion of the rubber molecular chains. Therefore, the interaction between molecular chains and particles with the matrix might be broken which leads to the decrement at the initial field modulus. The magneto-induced modulus of the MRE 45° sample at strain amplitudes of 0.01%, 0.1% and 1% are 0.73, 0.69, and 0.46 MPa, respectively. The phenomenon that occurred is due to the increment of the distance among particles with the increase of strain amplitude. Therefore, it resulted in the decrement of the interactive force between particles hence lower the magneto-induced modulus. This finding agrees with findings from previous studies [21,36].

**Figure 14:**
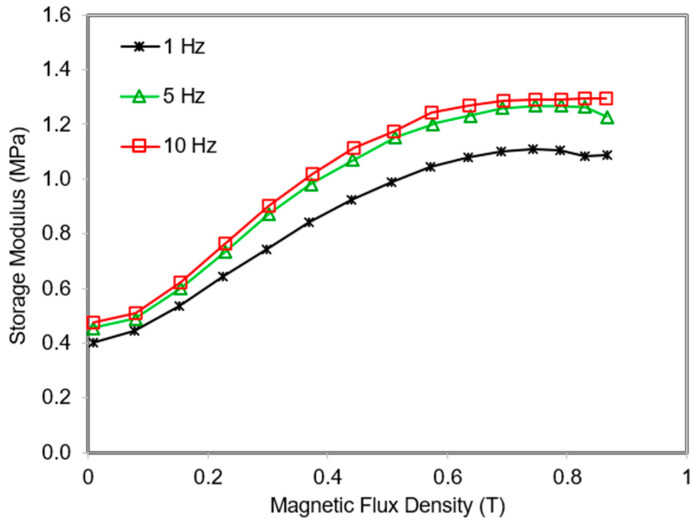
MRE 45° at various frequencies as a function of magnetic swept.

On the other hand, the influence of the ramped magnetic flux on the shear storage modulus of MREs with different frequencies was evaluated. The motion of the polymer molecular chain is frequency-dependent, therefore the MR effect of MRE is also frequency-dependent [21]. The MR effect of MRE 45° at 1, 5, and 10 Hz is shown in Figure 14. It can be noted that the initial-field modulus increases with the increment of the test frequency. By increasing the frequency, the motion of MRE 45° molecular chains cannot withstand the external stimuli, so molecular chains tend to be rigid and increased the value of the initial-field modulus. Meanwhile, all samples exhibited an increase in storage modulus as the intensity of the magnetic field increased. Attributed to the presence of a magnetic field, the attraction of the CIPs leads to the increased stiffness of MRE 45° and simultaneously enhanced the corresponding storage modulus. Nevertheless, even though the storage modulus increased as the magnetic field increased, the initial field modulus also increased, resulting the magneto-induced modulus to be the same value of 1 MPa although the samples were conducted at different frequencies. This value reveals that the magneto-induced modulus is independent of the test frequency; however, the relative MR effect decreases with increasing the test frequency due to the high value of the initial-field modulus.

## 4. Conclusions

In this study, MRE samples using an electromagnetic device with a new configuration mold with orientation of 0°, 45° and 90° were successfully fabricated, and the overall properties, related to the morphological observation, magnetic and rheological properties were experimentally investigated. It has been shown from the morphological analysis that the CIPs are randomly dispersed for isotropic MRE and assembled into chain-like structures for anisotropic MRE. More specific results achieved from this work are summarized as follows.

(a) The new configuration of magnetic flux mold for curing device produces 0.315 T through simulation with uniform distribution of magnetic flux flow across the MRE sample.
(b) The magnetic mold was inserted in the magnetic coil without an air gap to evade magnetic flux losses. The magnetic molds have functioned as guidance for the concentrated magnetic flux to flow parallelly from top to bottom parts that passing through the MRE sample, which was sandwiched between the mold’s parts for enhancing the alignment of CIPs in the MRE.
(c) The orientation of mold does alter the MR effect of MRE with the order of the storage modulus or MR effect versus orientations is 45° > 0° > 90° with 45° orientation achieved the highest relative MR effect of 909% and 1.01 MPa for magneto-induced modulus. It is interesting to note that the finding exceeds the result from the literature that obtained 0.6 MPa magneto-induced modulus based on 70 wt.% CIPs.
(d) Relative MR effect for MRE for mold orientation at 0°, 90° and isotropic distribution exhibited of 646%, 433% and 343%, respectively.
(e) Plasticization is an effective method to improve the MR effect. Without the SO, the relative MR effect obtained was only 173% with 0.45 MPa magneto-induced modulus for MRE 45°. Meanwhile, the relative MR effect is dependent on the test frequency and strain.

The resultant MR effect of the MRE with 45° mold oriented is much larger under shear deformation due to displacement of CIPs initiated additional shear stress into the matrix causing an increased in stress concentration of the matrix and between the CIPs, consequently increased the stiffness of the whole MRE. As a final note, the proposed MRE can be beneficial in industrial applications where they require a high range of stiffness, particularly under the shear deformation.

## Figures and Tables

**Table 1:** Input parameter of the components in the curing device of anisotropic MRE to be run in Finite element method magnetic (FEMM).

Component Name	Material
Mould (0°, 45° and 90°)	Mild steel
Steel Cover	Mild steel
Electromagnetic Coil	Copper (18 AWG (2270 turns))
Casing	Mild Steel
Bobbin	Mild Steel
Separator for 45° and 90° mold	Non-magnetic
MRE sample	Magnetic and Non-magnetic

**Table 2:** Magnetic parameters from the magnetization curves.

MRE Orientation (°)	*H_c_* [Oe]	*M_s_* [emu/g]	*M_r_* [emu/g]
0	8.00	141	0.27
45	8.30	145	0.29
90	7.52	135	0.26
isotropic	7.32	144	0.28

**Table 3:** The initial storage modulus (G_0_), the magnetic-induce modulus (∆G) and the MR effect of MRE samples with different mold orientations.

Sample	G_0_ (MPa)	G_max_ (MPa)	ΔG (MPa)	Relative MR Effect (%)
0°	0.12	0.92	0.79	646
45°	0.11	1.12	1.01	918
90°	0.09	0.48	0.39	433
isotropic	0.08	0.37	0.29	343

**Table 4:** The initial storage modulus (G_0_), the magnetic-induce modulus (∆G) and the MR effect of MRE 45° with and without plasticizer.

Sample	G_0_ (MPa)	G_max_ (MPa)	ΔG (MPa)	Relative MR Effect (%)
Without plasticizer	0.26	0.71	0.45	173
With plasticizer	0.11	1.12	1.01	918

## Data Availability

Not applicable.
